# Daring and Distress: Insights on Adolescent Risk Taking and Difficulties in Emotion Regulation from a Network Analysis Perspective

**DOI:** 10.3390/brainsci13091248

**Published:** 2023-08-26

**Authors:** Luca Cerniglia, Silvia Cimino, Renata Tambelli, Marco Lauriola

**Affiliations:** 1 Faculty of Psychology, International Telematic University Uninettuno, 00186 Rome, Italy; 2 Department of Dynamic, Clinical and Health Psychology, Sapienza, University of Rome, 00185 Rome, Italy; silvia.cimino@uniroma1.it (S.C.); renata.tambelli@uniroma1.it (R.T.); 3 Department of Social and Developmental Psychology, Sapienza University of Rome, 00185 Rome, Italy; marco.lauriola@uniroma1.it

**Keywords:** risk-taking behavior, self-harm, emotion regulation difficulties, personality, attachment styles, maladaptive psychological functioning, adolescent behavior, network analysis

## Abstract

We explored the interrelationships between risk-taking and self-harm in typically developing adolescents by examining various contributing factors, such as personality traits, difficulties in emotion regulation, attachment styles, and maladaptive psychological functioning. A sample of 234 Italian adolescents completed the Risk-Taking and Self-Harm Inventory for Adolescents (RTSHIA), the Risk-Taking Questionnaire (RT-18), the Difficulties in Emotion Regulation Strategies (DERS), the State Adult Attachment Measure (SAAM), and the Youth Self-Report (YSR). Network analysis was used to visualize and describe the interdependencies among the variables. Risk-taking behaviors were strongly linked to rule-breaking, aggression, and risk propensity, while self-harm behaviors were connected to limited access to emotion regulation strategies and thought problems. Centrality indices indicated that variables such as anxiety/depression, limited access to emotion regulation strategies, and rule-breaking had a high influence within the network. This study provided a comprehensive understanding of the nomological network of risk-taking and self-harm behaviors among adolescents. It highlighted the relative importance of factors such as emotion regulation difficulties and maladaptive psychological functioning in influencing these behaviors. The findings could inform psychological interventions and prevention strategies targeting adolescents at risk for engaging in risk-taking or self-harm behaviors.

## 1. Introduction

Engaging in potentially dangerous activities is a common feature of adolescence, as young people navigate their way toward independence and adulthood [1,2]. According to Steinberg [3,4], adolescents are more likely to engage in risky behaviors compared with adults or children because of a delay between the onset of puberty, which makes them crave excitement, and the gradual maturation of their cognitive control system, which enables adolescents to control these urges. Neurobiological studies have extended our understanding of adolescent risk-taking by showing that the reward-related brain circuitry matures sooner than the cognitive control-related circuitry [5]. Although all risk-taking behaviors share some commonalities in their outward manifestations, there are also fundamental differences between the underlying psychological processes, which means that each is influenced by a variety of individual factors and motivations in addition to developmental factors. For example, some forms of risk-taking are typically associated with personality characteristics, cognitive biases, and deployment of emotion regulation strategies [6,7]. Other forms of risk-taking are best understood as consequences or manifestations of adolescent psychopathology [8,9].

A key distinction is proposed between risk-taking (RT) and self-harm (SH), two categories that ostensibly differ in terms of intended goals, the social context in which they are typically carried out, adaptiveness for normative development, and underlying motivation [10]. Regarding intent and outcome, SH is often deliberate and planned [11]. In contrast, injuries and harm can occur as unintentional consequences of RT, such as reckless driving and dangerous challenges, often due to impulsive choices or disregard for future consequences [12]. Additionally, SH typically occurs when the adolescent is alone and socially isolated [13], whereas RT often takes place in public situations and is embedded in social dynamics and group behaviors [14,15]. Another fundamental difference is that RT has some degree of adaptive value as it contributes to autonomy and experimentation within certain limits [16,17], whereas SH is regarded as maladaptive. Even in small amounts, SH signals an adolescent’s inability to cope with negative emotions and difficult situations [18]. Therefore, while RT is often motivated by excitement and euphoria, SH is driven by emotional distress and likely mediated by difficulties in emotion regulation.

### 1.1. The Assessment of Risk-Taking and Self-Harm in Adolescents

The Risk-Taking and Self-Harm Inventory for Adolescents (RTSHIA) [19] is a self-report measure devised to assess adolescent RT and SH in community and clinical settings. The RTSHIA consists of 27 items covering exposure to situations or undertaking behaviors that could end in injury or loss to the individual. For example, RT items vary from minor actions, such as smoking cigarettes or engaging in potentially dangerous hobbies, to severe activities, like gang violence or exposing oneself to the risks of sexual promiscuity. On the other hand, SH items range from innocuous behaviors, like picking at wounds or pulling one’s hair out, to more severe actions, like overdosing or attempting suicide.

Previous research has shown that RT and SH emerged as distinct factors and are associated with different sets of individual factors. For example, Vrouva and colleagues [19] found that RT was more closely related to positive emotional states, sensation-seeking, and impulsivity, while SH was found to have a stronger association with negative emotional states and psychopathology, such as depression, anxiety, and suicidal tendencies. In addition, RT had stronger associations with external factors such as peer influence, family discord, and childhood abuse. In comparison, SH was more closely associated with internalizing factors, such as negative self-perception, body dissatisfaction, and uncertainty about social interactions and relationships.

The two-factor structure and high reliability of the RTSHIA were confirmed in the Portuguese language version [20]. The same study also found significant differences based on sex, with boys engaging in more RT and girls reporting more SH behaviors. The study also revealed that younger adolescents exhibited fewer RT and SH behaviors compared with older adolescents, and they were higher during adolescence than in preadolescence or adulthood. Xavier and colleagues [20] reported significant associations between SH and greater negative affect, lower positive affect, more daily and peer hassles, as well as victimization. These findings aligned with current empirical literature on risky behaviors in adolescence. For example, Reichl and Kaess [21] found SH to be an observable symptom of underlying emotional problems that can be a sensitive marker for the early detection of developmental trajectories of suicidal behavior and mental health problems.

The RTSHIA has recently undergone validation for use with Italian adolescents [22]. The findings of the study indicated that the factor structure of the scale aligned well with the English and Portuguese versions, suggesting high cross-cultural consistency of separate RT and SH factors. Additionally, the concurrent validity of the RTSHIA was supported by correlations with other questionnaires. One notable result was the identification of a common emotion regulation deficit underlying both RT and SH behaviors, specifically, the lack of emotional awareness. This finding indicated that difficulties in recognizing and understanding emotions contributed to the expression of both types of behaviors among adolescents. Furthermore, the study revealed that SH was associated with internalizing and externalizing psychopathology, indicating a broad range of psychological and behavioral difficulties. In contrast, RT was primarily associated with externalizing psychopathology, which encompasses behaviors such as aggression, impulsivity, and rule-breaking.

### 1.2. Personality Traits

Research has shown that individuals with certain personality characteristics are more likely to engage in risky behaviors than others. Sensation-seeking aspects, such as thrill and experience-seeking, were more strongly linked to taking recreational and social risks that trigger emotional arousal [7,23,24]. Instead, disregard for potential consequences and a lack of self-control have been related to risk-taking in the areas of ethics, health, safety, gambling, and finances [10,25,26]. Among the Big Five, conscientiousness and agreeableness had established links with risk aversion, while extraversion and openness to experience were found to be linked with risk-seeking [27,28,29]. RT was negatively correlated with aspects of neuroticism like worry and anxiety, while other aspects like anger and sadness encouraged it [7]. Furthermore, studies have found that individuals with certain personality disorders, such as borderline personality disorder, are at increased risk for engaging in SH, such as cutting and suicidal attempts [30].

### 1.3. Emotion Regulation

Emotion regulation refers to a broad set of abilities with which individuals can alter the range, intensity, and duration of their emotional experience [31]. These abilities, which are importantly influenced by genetic characteristics and the quality of the early interactions with significant caregivers [32,33,34], are crucial for maintaining adolescent physical and psychological well-being and gradually develop across the lifespan [35]. In the transition from childhood to adolescence, crucial skills related to monitoring and evaluating emotional states, such as executive functions, emotional awareness, and cognitive complexity, increase in maturity, resulting in better emotion regulation abilities [36]. However, it is only during late adolescence and emerging adulthood that the prefrontal cortex fully develops, allowing for the complete utilization of complex and flexible strategies to regulate emotions [37,38].

Adolescents, facing challenges in effectively managing intense emotions, may resort to engaging in potentially harmful behaviors. For example, difficulties in emotion regulation have been found to be associated with a higher involvement in substance abuse, unsafe sexual practices, and reckless driving [30,39]. Hence, there is a widespread belief that adolescents with underdeveloped emotion regulation skills may use RT as the only available strategy to regulate their negative emotions, pursue excitement, or impulsively act upon their emotions without fully considering the potential consequences [40].

Similarly, difficulties in emotion regulation have been associated with SH, including cutting, burning, and self-poisoning [41]. Research suggested that adolescents who engage in self-harm behaviors may do so as a way of coping with intense negative emotions, particularly feelings of distress or emotional pain [8,9]. Difficulties in emotion regulation may also make it difficult for adolescents to regulate their emotional responses to stress, which can further increase their risk for self-harm behaviors [11]. Recently, the National Institute of Mental Health (NIMH) stated that difficulties in emotion regulation can be drivers of suicidal and self-harm behaviors and that cognitive therapy is effective in strengthening skills that lead to improved emotion regulation to reduce suicide risk in youth [42].

### 1.4. Attachment

Secure attachment typically fosters the development of healthy emotion regulation skills [43,44]. Individuals with secure attachment feel safe and supported, and this makes them effectively regulate emotions and seek comfort during times of distress. On the other hand, insecure attachment, characterized by inconsistent or insensitive caregiving, can impede the development of emotion regulation abilities, resulting in difficulties in managing negative emotions. The intricate relationship between emotion regulation, attachment, and risk-taking highlights the importance of considering these factors in understanding human behavior and well-being. Recognizing these connections can inform interventions aimed at enhancing emotion regulation skills, promoting secure attachment, and mitigating the risk of engaging in harmful behaviors.

### 1.5. The Present Study

Considering the numerous psychological processes that underlie different types of risk-taking [10,11,12,13,14,15,16,17], the present study aims to enhance our understanding of the nomological network associated with RT and SH. A nomological network is a conceptual map that illustrates the connections between a target construct, such as RT or SH in our study, and other pertinent constructs—such as personality traits, emotion regulation difficulties, and maladaptive behaviors—that the literature designates as crucial for characterizing the target constructs.

While previous research has indicated an association between RT and SH with distinct individual difference factors [19,20,22], these conclusions were supported using bivariate correlation analyses. Such analyses only explore the relationships between pairs of variables, disregarding redundancy and overlap among multiple variables. For instance, Vrouva and colleagues [19] reported several significant bivariate correlations between SH and RT scores and 27 psychological disturbances. However, this bivariate approach hindered the assessment of specific psychological disturbances that uniquely contributed to heightened tendencies for RT and SH. In fact, the zero-order correlations highlighted in that study could have been redundant and failed to account for the influence of intercorrelated disturbances, potentially leading to misleading interpretations in the presence of substantial overlap of psychological symptoms. Similarly, Valle and colleagues [22] identified significant correlations between SH and four out of six emotion regulation difficulties. Yet again, the zero-order correlations impeded the assessment of which difficulty uniquely explained an increased inclination toward self-harm [19,20,22].

Different from previous research, the present study uses a network analysis approach, which can provide a more comprehensive and nuanced understanding of the nomological network compared with bivariate analyses [45]. Indeed, a network analysis is based on partial correlations, which can reveal the unique relationship between RT and SH and other key variables in the network after removing their overlap or redundancy. This is crucial for parsing genuine direct associations from spuriously inflated ones, and it is highly recommended for studying complex relations among highly comorbid or overlapping constructs [46].

Interpreting a large matrix of partial correlations can be challenging when attempting to understand the nomological network of RT and SH. In contrast, network analysis offers a convenient visual representation to illustrate the connections between the target constructs and key factors like personality traits, emotion regulation difficulties, and maladaptive behaviors. This visualization makes it easier to grasp the overall structure of the nomological network, highlighting clusters, hubs, and paths that might not be evident from simple correlations. Indeed, pinpointing the key elements within the network plot can provide insight into the main factors influencing RT and SH among adolescents and serve as potential targets for psychological intervention [47,48]. This outcome holds promise for mitigating the prevalence of RT and SH among adolescents.

In general, we expected RT to be uniquely associated with positive emotional states, sensation-seeking, impulsivity, signs of externalizing psychopathology, and difficulties in regulating emotions at a behavioral level. In contrast, we expected SH to be uniquely associated with negative emotional states, signs of internalizing psychopathology, and a lack of emotional awareness.

## 2. Materials and Methods

### 2.1. Participants

We recruited 234 Italian adolescents (93 girls, 140 boys, and 1 undisclosed gender) from secondary schools in Rome, with informed written consent obtained from parents or directly from participants if they were over 18 years of age. A portion of the sample in this study participated in cognitive tasks on separate occasions as part of a different research investigation. The outcomes and findings from that specific study have been published elsewhere [49]. Before recruitment, a psychologist introduced this study’s general aim to the school principal and parents. The sample’s age ranged from 13 to 19 years (M  =  15.70; SD  =  1.22), with no statistically significant difference between boys and girls (t-value  =  0.32; *p*  =  0.746). Based on general knowledge about the income and social prestige typically associated with certain professions, we broadly classified the socio-economic level of families according to the occupations of the parents. Families in the lower level (N = 80, 34%) included those where one or both parents held professions requiring lower levels of formal education and skill, often involving manual labor, routine tasks, or service roles with low societal prestige (e.g., housewife, bricklayer). In upper-level families (N = 53, 23%), one or both parents had achieved a professional role that required advanced degrees or specialized skills (e.g., doctors, engineers, lawyers, or high-ranking corporate roles). The remaining families (N = 101, 43%) were classified as middle-level, in which the highest professional role of one or both parents required a moderate level of education and skill (e.g., technical, skilled trades, administrative roles). The ethical committee for psychological research in the Department of Dynamic and Clinical Psychology approved this study (Prot. n. 0000018, 9 January 2019).

### 2.2. Instruments

#### 2.2.1. Risk-Taking and Self-Harm Inventory for Adolescents (RTSHIA)

The RTSHIA [22] is a 27-item self-report scale. Because two factors emerged during questionnaire development, separate RT and SH scores were developed and validated for use in community and clinical settings. All items referred to adolescent life history, and participants were asked to rate the frequency of each item using a 4-point scale (from 0 = never to 3 = many times). In the present study, we scored the items according to RT and SH subscales, with higher total scores indicating more risk-taking or self-harm. The Cronbach’s α in the current study were 0.91 and 0.84 for self-harm and risk-taking, respectively.

#### 2.2.2. Risk-Taking Questionnaire (RT-18)

The RT-18 [50] consists of 18 items that were taken from existing personality questionnaires assessing risk-related traits (e.g., the Temperament and Character Inventory, Zuckerman Kuhlman Personality Questionnaire, and Impulsiveness–Venturesomeness–Empathy Questionnaire). The RT-18 questionnaire measures two distinct factors for risk-taking behavior, which are represented by two subscale scores: risk-taking propensity—hereafter referred to as risk propensity (RP)—and risk assessment (RA). Nine RP items assess the propensity to engage in high-arousal recreational forms of risk-taking, (e.g., I sometimes do “crazy” things just for fun). The label RP is used to describe a personality inclined toward risk-taking, driven by the pursuit of sensation-seeking behaviors. Nine RA items measure the tendency to act impulsively versus reflectively in everyday situations (e.g., I often get into a jam because I do things without thinking vs. I like to think about things for a long time before I make a decision). The label RA is used to describe a personality inclined toward risk-taking due to impulsive actions and a tendency to disregard future consequences. Participants respond to each item with either “yes” or “no,” and receive 0 or 1 point for each item, with a total score ranging from 0 (minimum risk-taking/risk assessment) to 9 (maximum risk-taking/risk assessment). In the current study, Cronbach’s alpha coefficients for the RT and RA subscales were 0.71 and 0.75, respectively.

#### 2.2.3. Difficulties in Emotion Regulation Strategies (DERS)

The DERS [51] is a self-report questionnaire developed to assess a range of emotion regulation problems. It encompasses 36 Likert-type items, asking respondents how they relate to their emotions (from 1 = almost never to 5 = almost always). The DERS yields the following subscale scores: (1) lack of acceptance of the emotional responses (or non-acceptance), (2) difficulty controlling impulsive behaviors and behaving in accordance with desired goals (or goals), (3) limited access to emotion regulation strategies (or strategies), (4) lack of control when experiencing intense emotions (or impulse), (5) difficulties recognizing emotions (or clarity), and (6) limited awareness and understanding of emotions (or awareness). Higher scores reflect more emotion regulation problems. The Cronbach’s α in the current study were 0.82, 0.75, 0.83, 0.81, 0.78, and 0.69, respectively for non-acceptance, goals, strategies, impulse, clarity, and awareness.

#### 2.2.4. State Adult Attachment Measure (SAAM)

The SAAM [52] was originally created to assess temporary fluctuations in adult attachment styles in response to priming manipulations. Subsequent studies revealed that the SAAM is aligned with standard trait attachment measures [53] and can be effectively used in adolescent samples [54]. The SAAM is a 21-item self-report scale asking people how they feel “at the moment” using a seven-point Likert scale (from 1 = disagree strongly to 7 = agree strongly). It provides three different assessments of attachment: security (e.g., I feel relaxed knowing that close others are there for me right now), anxiety (e.g., I really need to feel loved right now), and avoidance (e.g., The idea of being emotionally close to someone makes me nervous). The Cronbach’s α in the current study was 0.84, 0.83, and 0.78, respectively for security, anxiety, and avoidance.

#### 2.2.5. Youth Self-Report (YSR)

The YSR [55] is one of the most widely used self-report measures for the assessment of emotional and behavioral problems in adolescents. It is composed of 112 problem items, each scored on a 3-point scale (0 = not true, 1 = somewhat or sometimes true, 2 = very or often true). The YSR yields eight subscales: anxious/depressed, withdrawn, somatization, social problems, thought problems, attention problems, rule-breaking, and aggression. Moreover, withdrawn, somatization, and anxious/depressed together can be summarized in a broad “Internalizing” dimension (31 items), whereas rule-breaking and aggressive behaviors constitute an “Externalizing” dimension (32 items). Higher scores on YSR scales indicate more maladaptive psychological functioning. The Cronbach’s alphas in the present study were 0.85, 0.78, 0.80, 0.79, 0.82, 0.69, 0.83, and 0.82 for anxious/depressed, withdrawn, somatization, social problems, thought problems, attention problems, rule-breaking, and aggression, respectively.

### 2.3. Statistical Analysis

#### 2.3.1. Parametric Assumptions and Handling Missing Data

For each study variable, total scores were computed (descriptive statistics are listed in Table 1). Because of sporadic missing values (up to 6 cases per variable), we carried out a missing value analysis. Little’s MCAR test (χ2 = 101.50, df = 90, *p* = 0.191) was not significant, showing that the missing data pattern was completely random. Under this assumption, statistical analyses can be safely carried out using listwise deletion or full information maximum likelihood imputation (FIML). The Shapiro–Wilk test was significant for all variables, indicating that normality was violated. However, the data distribution was only slightly asymmetrical (i.e., skewness between −1 and 1) for most variables, raising concerns only for self-harm and social problems (skewness = 2.25 and 1.11, respectively). Likewise, the Kurtosis was within the acceptable range (i.e., kurtosis between −3 and 3) for all variables, except self-harm (kurtosis = 5.50), indicating that extreme values were not very different from those expected according to a normal data distribution. Nonparametric Spearman correlations were used to check significant associations among study variables.

#### 2.3.2. Network Analysis

We used the bootnet and qgraph packages for R to estimate and visualize a network model including risk-taking variables, difficulties in emotion regulation, attachment, and YSR syndrome scales [56]. Given the relatively high number of variables in the network (n = 21), we applied the EBIC–Glasso algorithm, which returns a parsimonious network model with the smallest number of parameters explaining the covariation structure in the data. To cope with violations of normality, a non-paranormal transformation of the variables was also applied. This transformation converted the observed data to approximate a multivariate normal distribution, allowing for an accurate estimation of the correlation structure [57].

The ability of a network structure to resist change as research participants are gradually excluded from the sample was used to test the network stability. Following Epskamp and colleagues [58], we calculated the correlation stability coefficient (CS), an index for network stability calculated as the correlation between centrality indices resulting from the original network and those resulting from a network estimated on a subset of cases (usually 70%). The CS should not be lower than 0.25 and preferably above 0.5 to ensure network stability. A second desirable property of a network structure is edge accuracy, that is, the extent to which the connections between nodes can be considered reliable. Taking a nonparametric approach, edge accuracy was estimated using 95% bootstrap CIs.

The network diagram consists of nodes (i.e., variables) and edges (i.e., connections between nodes), which represent the strength of an association between variables by varying thickness. Nonzero edges can be thought of as partial correlations or net effects that account for all other network variables. In addition to visual inspection, we assessed the importance of network nodes using centrality statistics, which help describe how strongly changes in a network node are associated with a change in the remaining nodes. Strength centrality quantifies the number of edges a node has with other nodes and represents how likely it is for a particular node to activate other nodes in the network. Betweenness centrality measures a node’s importance in terms of connectivity and is determined by how much it interposes itself among other nodes in the network. Finally, closeness centrality is related to the average distance between a node and others in the network and represents the level of dependence of a node from other nodes.

Community detection allows researchers to identify clusters of nodes that are more strongly connected to each other than they are to other nodes in the network. These clusters can be used to better understand the structure of the network and the relationships between different variables. For example, in a network of symptoms of a mental disorder, community detection could be used to identify groups of symptoms that are often co-occurring. The igraph package for R was used for community detection purposes. Specifically, we used the Spinglass algorithm, which is one of many algorithms that allow researchers to identify highly coherent clusters. This algorithm works by optimizing a function that rewards edges within a community and penalizes edges between communities. The algorithm stops when the function is minimized, which means that it has found a set of clusters that are as cohesive as possible [59]. However, because the Spinglass algorithm uses a random initial state, explores the network structure following a random walk, and uses a stopping criterion that is based on a random variable, the number of detected clusters is subject to some degree of variability.

## 3. Results

Before studying the correlations between the variables, we examined gender and age differences in risk-taking variables, difficulties in emotion regulation, attachment scales, and YSR syndrome scales. The gender differences are reported in Table 2. Boys exhibited higher RT scores compared with girls, although the difference did not reach statistical significance using the Mann–Whitney test. However, a significant gender difference was found in SH, with males reporting lower scores than females. Similarly, gender differences were evident in goals, clarity, and strategies. In all cases, girls reported higher difficulties in emotion regulation than boys. Additionally, girls exhibited higher levels of attachment anxiety compared with boys and scored higher than boys in anxious/depressed YSR domains. Finally, girls reported more somatic complaints and thought problems than boys.

Table 3 shows the age differences. Early adolescents (under 16 years) reported lower levels of risk-taking compared with late adolescents (over 16 years old). This difference was highly significant. However, no significant age differences were found in self-harm and risk assessment. Late adolescents showed higher scores in non-acceptance and impulse control compared with early adolescents, with statistically significant age differences. In the domain of anxiety, late adolescents exhibited higher scores compared with early adolescents, with a statistically significant age difference. Similarly, late adolescents scored higher in anxious/depressed symptoms compared with early adolescents, with a highly significant age difference. Regarding maladaptive psychological functioning, late adolescents scored higher in withdrawn behavior, somatic complaints, thought problems, and aggression compared with early adolescents, with a statistically significant age difference.

Zero-order correlations among risk-taking variables, difficulties in emotion regulation, attachment, and YSR syndrome scales are reported in Table 3. In terms of effect size, the correlations within each set of variables were moderate to high according to Cohen’s standards. For example, awareness was found to be strongly related to strategies and clarity and moderately to goals and impulse. Attachment security was more aligned with avoidance than anxiety. The YSR syndrome scales were highly intercorrelated, with effect sizes ranging from around 0.30 (for rule-breaking with depression/anxiety and withdrawal) to around 0.70 (for depression/anxiety with internalizing symptoms such as withdrawal, somatic problems, and social problems). Both RT and SH showed positive correlations with variables such as impulse, awareness, thought problems, and attention problems, with effect sizes ranging from medium to large. Attachment security was instead negatively associated with both RT and SH. Two major differences emerged in the correlation pattern for RT and SH with the other variables. On the one hand, RT was more strongly associated with rule-breaking, aggressive, risk propensity, and risk assessment compared with SH. On the other hand, SH was more strongly associated with non-acceptance, goals, strategies, clarity, anxious/depressed, withdrawn, somatic, social problems, attachment anxiety, and avoidance. RT was not even significant with goals, clarity, and attachment anxiety.

**Figure 1:**
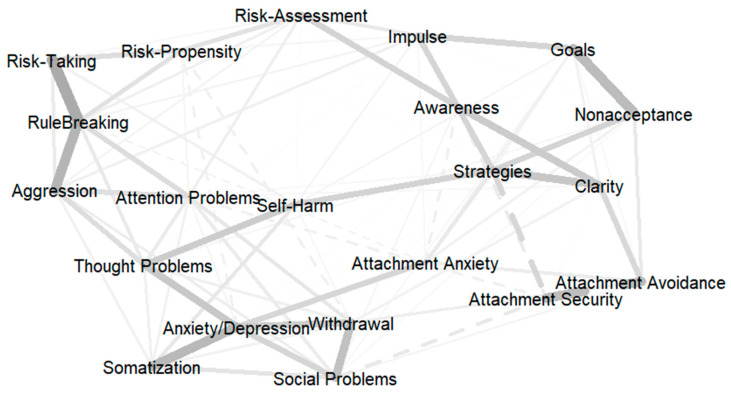
Network plot illustrating the interdependencies among risk-taking, self-harm, and related factors in typically developing adolescents. Edge thickness represents the weight of the relationship, with thicker edges indicating stronger connections. Dotted edges represent negative weights, indicating an inverse relationship, while solid edges represent positive weights, indicating a direct relationship.

While a correlation analysis primarily focuses on assessing the strength and direction of relationships between pairs of variables, a network analysis aims to visualize and understand the complex interdependencies among multiple variables simultaneously. The estimated network of risk-taking variables, difficulties in emotion regulation, attachment, and YSR syndrome scales consisted of 21 nodes and 97 non-zero edges out of 210 edges, with an overall density of 45.71%. All variables had at least one close relationship with the other variables. Before interpreting the network plot (reported in Figure 1) and centrality indices for each variable (reported in Table 3), we tested the network’s overall stability using the central stability (CS) coefficient. After 5000 bootstrap replications, we obtained a CS equal to 0.52 for both edge and strength accuracy, which is satisfactory according to current standards (i.e., CS > 0.50) [58]. Therefore, the edge weights and the order of node strength can be interpreted with a good degree of confidence.

As shown in Figure 1, RT and SH were in separate regions, and each was interconnected with different nodes. According to edge thickness, RT was strongly linked to rule-breaking, aggression, and risk propensity. Instead, SH was strongly tied to emotion regulation strategies and thought problems. Other thick edges in the network interconnected the six emotion regulation difficulties, internalizing YSR scales (such as anxiety/depression, withdrawal, and somatization), and attachment security with attachment avoidance (in a negative direction). Edge betweenness centrality is a network index used to identify the importance of edges in the network in terms of connecting different nodes in the plot based on the total number of shortest paths that pass through them. Accordingly, the two most important edges in the network were bridging SH to strategies and thought problems (with centrality values of 42 and 37, respectively). Other relevant bridges revolved around aggression, linking it to thought problems and rule-breaking (in both cases the centrality value was 26). The connection between RT and aggression in terms of edge betweenness was the tenth most important (with a centrality value of 16).

Table 4 reports the node centrality indices. Strength centrality is a measure used to quantify the influence or importance of nodes within a network based on how tightly each individual node is directly connected to other nodes. Regarding strength centrality, network nodes such as anxiety/depression, strategies, and rule-breaking were the three strongest variables in the network, uniquely accounting for the largest proportion of variance in the surrounding nodes. Other relatively strong nodes were the other YSR scales (except somatization). RT and SH were at the bottom of the strength centrality ranking. Because strength centrality can be interpreted in terms of how likely it is for a particular node to activate other nodes in the network (Figure 1), it follows that YSR syndrome scales were more likely to activate the surrounding nodes to a larger extent than RT or SH. This finding is consistent with the view that risk-taking behaviors of any kind are likely the distal consequences of personality, emotion dysregulation, and psychopathology processes. For example, rule-breaking can lead to RT as well as aggression and attention problems, which might have severe consequences for adolescents. Strategies can also lead to SH. Despite being the strongest node in the network, anxiety/depression had neither RT nor SH among the surrounding nodes.

Betweenness centrality is used to identify the nodes that act as important bridges or intermediaries within a network. Indeed, nodes with higher betweenness centrality can be thought of as critical points in the hypothetical process modeled by the whole network. As shown in Table 3, strategies were the most influential node in terms of betweenness centrality, followed by thought problems and SH. Interpreting the network plot in light of betweenness centrality, it appears that strategies and thought problems occupied a strategic position, having the greatest potential to mediate the relationships between emotion dysregulation difficulties (on the on hand) and maladaptive psychological functioning (on the other hand), exerting a significant impact on SH, which lied at the core of the network plot (Figure 1).

Strategies, thought problems, and SH were also among the top three nodes in terms of closeness centrality (Table 5). High closeness centrality indicates that a node is located at a relatively short average distance from other nodes, and thus much of its variance is accounted for by stronger nodes in the periphery. In certain network structures, closeness centrality and betweenness centrality can be highly correlated (r = 0.80 in the present study). When this occurs, nodes that are positioned as intermediaries, connecting different parts of the network, often have shorter average distances to other nodes. In the context of the present study, this means that strategies, thought problems, and SH were both traversed by a larger number of shortest paths (high betweenness) and had the potential to affect other nodes in the network quickly and easily (high closeness).

The Spinglass algorithm detected three to five communities, with a median value of 3, after 500 bootstrap replications. As reported in Table 3, the first community included RT, social problems, thought problems, attention problems, rule breaking, and aggressive. The second community included SH, attachment anxiety and avoidance, anxious/depressed, withdrawn, and somatic. Lastly, the third community included risk assessment, risk-propensity, non-acceptance, goals, strategies, and impulse.

Community 1 suggested that adolescents who tended to engage in RT behaviors, such as substance use, reckless driving, and fighting, tended to report maladaptive psychological functioning directed toward the external environment. For example, they might have experienced unusual thoughts or beliefs, such as being followed or having special powers, had difficulty following rules at home, at school, or in the community, and could be aggressive toward others, both verbally and physically.

Community 2 suggested that adolescents who tended to engage in SH behaviors, such as cutting or burning themselves, were also more likely to experience anxiety, depression, and withdrawal. Furthermore, high levels of attachment anxiety and avoidance co-occurred with both SH and the experience of emotional issues that are characterized by turning inward. For example, adolescents with high levels of attachment anxiety may be more likely to engage in self-harm to cope with their fear of abandonment. They may also be more likely to form relationships with people who are emotionally unavailable, which can reinforce their negative beliefs about themselves and their ability to be loved.

Finally, community 3 was characterized by difficulties with emotion regulation and risk-related personality characteristics. The cluster included five DERS subscales for non-acceptance, goals, strategies, and clarity plus risk-propensity (i.e., sensation seeking), risk assessment (i.e., impulsivity), and attachment security (as a protective factor). Accordingly, adolescents who had difficulty noticing their emotions, acknowledging and accepting their emotions, setting and achieving emotional goals, difficulty using effective emotion regulation strategies, and difficulty controlling impulsive behaviors in response to intense emotional arousal also tended to be less reflective in assessing the riskiness of certain behaviors and demonstrated a reduced sensitivity to potential dangers or risks, might have exceeded healthy limits, and the desire for new experiences and excitement can lead them to engage in risky behaviors to fit in or impress others.

## 4. Discussion

Using the RTSHIA, a research tool that assesses adolescent RT and SH in community and clinical settings, we aimed to investigate the unique associations between risk-related personality tendencies, difficulties in emotion regulation, attachment, and maladaptive psychological functioning in a sample of typically developing adolescents. Previous research suggested that RT and SH were associated with different sets of individual difference factors [19,20,22]. However, these findings were generally supported using correlation analyses, which focus on examining the relationship between pairs of variables, ignoring the interdependencies among multiple variables defining the nomological network of RT and SH. We also conducted a correlation analysis, which suggested two major differences in the correlation patterns of RT and SH. On the one hand, RT was more strongly associated with externalizing syndrome scales and a personality inclined toward risk-taking, driven by the pursuit of sensation-seeking behaviors [23,24,60] or due to impulsive actions and a tendency to disregard future consequences [10,25,26]. On the other hand, SH was more strongly associated with difficulties in emotion regulation and internalizing syndrome scales, thus aligning with previous studies suggesting that adolescents who engage in self-harm behaviors may do so as maladaptive coping with intense negative emotions directed toward the self [11,22,42].

The network analysis used in the present study offered a broader perspective by considering the interplay between multiple variables simultaneously and highlighting the unique contribution of specific variables to the nomological network of the two constructs [45,58,61]. At first glance, the network plot clearly revealed distinct topologies for RT and SH, with each located apart in the network and uniquely associated with different variables. For example, RT appeared relatively peripheral in the network and was linked the most to rule-breaking, aggression, and risk propensity. In contrast, SH was in the center of the plot and tied the most to limited access to emotion regulation strategies and thought problems. Collectively, these observations were overall consistent with our research hypotheses and the literature showing that personality factors may be more influential in predicting RT [7,24], while emotion regulation difficulties and maladaptive psychological functioning may be more significant predictors of SH [8,9,11,42].

The kinds of behaviors that defined RT in the RTSHIA included putting oneself in risky situations (e.g., traveling without a valid ticket, shoplifting) despite the possibility of getting caught, being suspended or dropping out of school, staying out late at night without informing parents, participating in gang violence or physical fights, possessing weapons, engaging in sexual promiscuity without precautions, getting drunk, using drugs, and smoking tobacco. These behaviors were more frequently reported by adolescents inclined to defy societal norms and regulations, engage in hostile or rough behaviors toward others, and seek novel, intense, and thrilling experiences. In typically developing adolescents, rule-breaking can create a sense of excitement, rebellion, and independence, leading them to disregard potential consequences or warnings associated with risky activities. They may view smoking as an act of defiance against health warnings, and in more severe cases, rule-breaking tendencies can contribute to involvement in criminal activities such as gang violence. Furthermore, unprotected sex and sexual promiscuity reflect rule breaking in the sense that they involve defying established rules regarding sexual behavior and responsible healthy practices.

In the RTSHIA, SH encompasses behaviors, such as cutting, burning, biting, hair-pulling, head-banging, hitting, using sharp objects and toxic substances to inflict pain, disordered eating, self-inflicting emotional harm, and suicidal ideation and attempts. We found that these behaviors were more frequently reported by adolescents who experienced thought problems and had limited access to emotion regulation strategies. Therefore, our study suggests that self-harm is more likely to occur in teenagers who exhibit unconventional, bizarre, or disconnected thoughts, engage in unconventional actions, hold false beliefs that persist despite evidence to the contrary, or demonstrate disorganization in expressing themselves clearly. Additionally, adolescents who struggle to utilize effective strategies for regulating their emotions, such as problem identification, reframing situations to reduce emotional impact, taking proactive steps, and expressing emotions in a healthy manner, may be more prone to experiencing emotional distress, including anxiety, depression, and anger. Furthermore, they may be more inclined to engage in unhealthy coping mechanisms, such as substance abuse or self-harm.

Central variables in network analysis are thought to suggest appropriate targets for psychological interventions [45,47], as modifying or addressing them may have a greater potential for producing widespread changes in the whole network [47,48]. For example, anxiety/depression, limited access to emotion regulation strategies, and rule-breaking had the highest strength centrality, suggesting that addressing these problems in adolescents can have the greatest impact on the surrounding nodes, including RT (because of its proximity to rule-breaking) and SH (because of its proximity to both anxiety/depression and limited access to emotion regulation strategies). Furthermore, betweenness centrality highlighted that limited access to emotion regulation strategies and thought problems were the main “bridges” that linked SH to all other variables in the network. Just as hub symptoms generate indirect effects on all the symptoms they are connected to [48], similarly, limited access to emotion regulation strategies and thought problems can mediate between a variety of emotion dysregulation difficulties, maladaptive psychological functioning, and SH [8,9,11,42].

Limited access to emotion regulation strategies includes items such as “I have trouble calming down when I’m upset”, “I have trouble finding ways to cope with my negative emotions”, and “I have trouble stopping myself from doing things that I know will make me feel worse” [51]. Adolescents who score high on this subscale may have learned that there is little one can do to regulate one’s emotions once experiencing negative affect. Our study suggests that psychological interventions should target these maladaptive beliefs to prevent SH in typically developing adolescents. For example, there are many effective strategies that can help you to improve your ability to regulate your emotions. For example, adolescents should be taught that numerous effective strategies exist to enhance their capacity for emotion regulation. Because thought problems were the second most important bridge to SH, preventive interventions should be oriented to adolescents who may experience temporary difficulties in distinguishing reality from fantasy and exhibit disorganized thoughts. Thought problems are not uncommon in community adolescents, with a prevalence of up to 7–8% among those aged 13 to 18 years [62], and—in our study—thought problems were uniquely associated with SH and other risk-taking behaviors. Given the relatively high prevalence, our findings emphasize the importance of addressing maladaptive beliefs related to emotion regulation, particularly among adolescents who are more prone to experiencing psychotic-like experiences.

Regarding the validity of the RTSHIA, we carried out a community detection analysis, which revealed that RT was part of the first community with social problems, thought problems, attention problems, rule-breaking, and aggression, suggesting that adolescents engaged in risk-taking behaviors tended to exhibit maladaptive psychological functioning directed toward the external environment. By contrast, SH was included in the second community with attachment anxiety and avoidance, anxious/depressed, withdrawn, and somatic symptoms. Communities in psychometric networks represent groups of variables that are closely interconnected within the network, providing insights into possible co-syndromes or comorbidities, while factors in exploratory factor analysis are thought to represent underlying latent causes that explain why observed variables share a significant amount of variance. Despite differences between the two approaches [61], our findings align with previous research showing that RT and SH are thought to be independent constructs [19,20,22]. More generally, the fact that RT and SH did not cluster together fosters conceptual distinctions between the two types of risky behaviors in terms of intended goals, the social context in which they are typically carried out, adaptiveness for normative development, and underlying motivation [10].

Except for clarity, the third community included all subscales from the DERS. This assessment tool stands as one of the most widely used self-report questionnaires for assessing deficits in emotion regulation. However, it is noteworthy that a longstanding debate exists concerning the factor structure of the DERS and the use of subscales versus a single composite score [63]. Not all investigations have confirmed the original six-factor structure. Some scholars [64] proposed a five-factor model, while others [63] posited the existence of a general emotion dysregulation factor. One might be led to believe that the third community identified in the present study may contribute to the larger debate on the structure of the DERS and that using a composite score instead of subscales may be more parsimonious. It is worth remembering, however, that in a network analysis framework, a community such as community 3 is not always synonymous with a latent variable or a composite measure representing a unified construct. For example, a dense community can arise from various factors, including shared themes, contents, or even methodological overlaps among variables. More conservatively, we believe that the third community suggests that a subgroup of DERS subscales shared unique interconnections within a specific context, such as the nomological network of RT and SH. Previous research has also found that a lack of emotional awareness is a common emotion regulation deficit underlying both RT and SH. However, our study found that limited access to emotion regulation strategies is more closely related to SH than RT. This discrepancy may be due to the multivariate approach used in our study, which allowed us to examine the complex relationships between these variables. In the study by Valle and colleagues, the bivariate correlations between RT and SH with awareness were 0.28 and 0.27, respectively. In our study, the corresponding coefficients were 0.17 and 0.21. These lower correlations were not divergent enough to suggest that awareness was less strongly related to RT and SH in our sample. However, when we examined the topology of the network plot and the centrality indices of network analysis, a different picture emerged. Limited access to emotion regulation strategies was found to be a central predictor of SH, while lack of emotional awareness was only indirectly related to RT behaviors (through sensation-seeking and impulsive tendencies measured using the RT-18). This suggests that limited access to emotion regulation strategies may be a more important factor in the development of SH than a lack of emotional awareness.

Before concluding is worth acknowledging the following limitations. First, and foremost, the sample size was relatively small for a network analysis. Conducting a power analysis for network models is challenging. Network type, density, and edge weights all play a role, and “ad-hoc” simulation studies are needed. Using a such simulation study, Constantin [65] discovered that sample sizes ranging between 250 and 350 generally yield sufficient observations to provide moderate sensitivity, high specificity, and strong correlation in edge weights for networks comprising 20 nodes. Our study involved 234 participants and 21 nodes. These features placed our study at the edges of the recommended minimum standards. Hence, it is possible that due to limited power, there could be statistically significant associations that we did not detect. Notwithstanding this limitation, our network model exhibited a sufficient stability, in line with current accuracy standards [58], regarding edge weights and the hierarchy of node strengths. Additionally, our findings are consistent with prior research on RT and SH in Italy, as well as with international samples. Relatedly, a second limitation concerns the generalizability of our findings across cultural contexts. Cultural influences can mold the way adolescents think, feel, and express themselves behaviorally. While our study centered on Italian adolescents, it is important to recognize that assuming universal applicability across cultures may be limited. Therefore, careful consideration is necessary when assessing the potential generalizability of our results. Although our findings align with previous research on British and Portuguese adolescent samples, we acknowledge the importance of conducting similar studies in different cultural settings to understand the broader relevance of our conclusions. Another limitation could be the inherent limitations in the explanatory power of correlations, a challenge not exclusive to our study but shared by many in our field. Our research is indeed descriptive in nature, aiming to outline patterns and associations within the intricate realm of adolescent behaviors. This limits the generalizability of our findings to dynamic contexts in which risk-taking and self-harm behaviors occur [66,67,68,69,70]. Longitudinal or intervention studies would offer a better understanding of the temporal order and causal mechanisms between the study variables.

## 5. Conclusions

Overall, our study contributed to the understanding of the complex relationships among risk-taking behaviors, difficulties in emotion regulation, attachment, and psychological symptoms in adolescents. The findings emphasized the importance of considering multiple factors in the assessment and intervention of risk-taking behaviors in this population. The network analysis approach provided a comprehensive framework to identify influential nodes and their interdependencies, shedding light on potential targets for intervention strategies. Strategies aimed at improving emotion regulation skills, addressing thought problems, and fostering secure attachment relationships may have a significant impact on reducing risk-taking behaviors and related psychological symptoms. Future research should further investigate these relationships longitudinally and explore potential causal pathways to inform prevention and intervention efforts targeted at reducing risk-taking behaviors and promoting healthy adolescent development.

## Figures and Tables

**Table 1:** Descriptive statistics and test of normality for the study variables.

	N	M	SD	Sk	K	W	*p*
1. Risk Taking	233	6.76	6.23	0.78	−0.42	0.89	<0.001
2. Self-Harm	233	5.34	8.07	2.27	5.62	0.69	<0.001
3. Risk Assessment	231	3.58	2.44	0.21	−0.97	0.95	<0.001
4. Risk-Propensity	233	5.21	2.4	−0.3	−0.79	0.95	<0.001
5. Non-Acceptance	233	12.8	5.85	0.56	−0.65	0.92	<0.001
6. Goals	233	13.17	4.76	0.51	−0.43	0.96	<0.001
7. Strategies	233	19.85	6.34	0.55	0.08	0.97	<0.001
8. Impulse	233	13.86	5.62	0.6	−0.42	0.95	<0.001
9. Clarity	233	12.34	4.57	0.58	−0.26	0.96	<0.001
10. Awareness	233	7.39	3.02	0.46	−0.6	0.95	<0.001
11. Security	229	34.79	9.19	−0.63	−0.32	0.95	<0.001
12. Anxiety	231	29.2	9.73	−0.17	−0.57	0.99	0.027
13. Avoidance	231	21.27	8.6	0.32	−0.31	0.98	<0.001
14. Anxious/Depressed	232	7.62	5.33	0.68	−0.11	0.95	<0.001
15. Withdrawn	232	4.6	3.47	0.67	0	0.94	<0.001
16. Somatic	232	4.59	3.89	0.91	0.57	0.91	<0.001
17. Social Problems	232	4.35	3.81	1.12	1.61	0.9	<0.001
18. Thought Problems	232	5.64	4.69	0.98	0.74	0.92	<0.001
19. Attention Problems	232	5.91	3.38	0.25	−0.31	0.98	<0.001
20. Rule Breaking	232	6.71	5.27	0.93	0.49	0.93	<0.001
21. Aggressive	232	9.35	5.67	0.64	0.16	0.96	<0.001

Legend: N = number of valid cases; M = mean; SD = standard deviation; Sk = skewness; K = kurtosis; W = Shapiro–Wilk statistic; *p* = *p*-value for W.

**Table 2:** Gender Differences in Risk-Taking and Self-Harm Behaviors and Other Psychological Variables.

	Boys		Girls		Student’s *t*-Test		Mann–Whitney Test	
	M	SD	M	SD	t	*p*	U	*p*
1. Risk Taking	7.43	6.66	5.76	5.41	2.01	0.046	5768	0.140
2. Self-Harm	4.31	7.31	6.88	8.92	−2.40	0.017	4936	0.001
3. Risk Assessment	3.38	2.52	3.87	2.30	−1.49	0.137	5608	0.102
4. Risk-Propensity	5.32	2.30	5.05	2.55	0.83	0.405	6131	0.449
5. Non-Acceptance	12.67	5.65	13.00	6.17	−0.42	0.676	6362	0.768
6. Goals	12.51	4.46	14.16	5.04	−2.63	0.009	5325	0.018
7. Strategies	18.89	6.15	21.31	6.38	−2.90	0.004	5160	0.007
8. Impulse	13.43	5.50	14.52	5.78	−1.45	0.149	5791	0.153
9. Clarity	11.61	4.34	13.45	4.71	−3.07	0.002	5047	0.004
10. Awareness	7.31	2.98	7.53	3.10	−0.54	0.588	6281	0.648
11. Security	35.25	8.91	34.11	9.61	0.92	0.361	5876	0.411
12. Anxiety	26.21	9.43	33.72	8.37	−6.19	<0.001	3348	<0.001
13. Avoidance	20.54	8.53	22.38	8.64	−1.60	0.112	5631	0.125
14. Anxious/Depressed	6.40	5.30	9.43	4.86	−4.40	<0.001	4032	<0.001
15. Withdrawn	4.05	3.39	5.43	3.44	−3.02	0.003	4906	0.002
16. Somatic	3.69	3.53	5.95	4.03	−4.50	<0.001	4218	<0.001
17. Social Problems	4.21	4.05	4.56	3.44	−0.68	0.494	5787	0.175
18. Thought Problems	5.23	4.99	6.26	4.14	−1.64	0.102	5198	0.011
19. Attention Problems	5.71	3.66	6.20	2.90	−1.08	0.278	5826	0.201
20. Rule Breaking	7.28	5.96	5.86	3.92	2.03	0.044	5974	0.328
21. Aggressive	9.43	6.35	9.24	4.52	0.26	0.798	6190	0.585

Legend: M = mean; SD = standard deviation; t = t statistic; U = U statistic; *p* = *p*-value.

**Table 3:** Age Differences in Risk-Taking and Self-Harm Behaviors and Other Psychological Variables.

	Age under 16		Age over 16		Student’s *t*-Test		Mann–Whitney Test	
	M	SD	M	SD	t	*p*	U	*p*
1. Risk Taking	4.83	5.16	8.05	6.56	−3.99	<0.001	4704	<0.001
2. Self-Harm	4.66	7.24	5.79	8.57	−1.05	0.293	6087	0.393
3. Risk Assessment	3.85	2.55	3.40	2.36	1.38	0.169	5794	0.208
4. Risk-Propensity	5.54	2.36	5.00	2.41	1.68	0.094	5680	0.097
5. Non-Acceptance	11.74	5.27	13.51	6.13	−2.27	0.024	5509	0.046
6. Goals	12.40	4.34	13.68	4.97	−2.02	0.044	5618	0.076
7. Strategies	18.95	6.11	20.46	6.45	−1.79	0.075	5577	0.064
8. Impulse	13.05	5.56	14.40	5.62	−1.80	0.073	5511	0.047
9. Clarity	11.94	4.34	12.61	4.71	−1.11	0.268	5961	0.275
10. Awareness	7.34	2.91	7.43	3.10	−0.21	0.835	6417	0.854
11. Security	35.20	8.08	34.53	9.87	0.54	0.593	6214	0.934
12. Anxiety	27.21	9.60	30.52	9.62	−2.56	0.011	5142	0.012
13. Avoidance	20.46	7.49	21.81	9.26	−1.17	0.242	5863	0.286
14. Anxious/Depressed	6.20	4.78	8.56	5.48	−3.37	<0.001	4761	<0.001
15. Withdrawn	4.01	3.21	5.00	3.59	−2.14	0.033	5432	0.039
16. Somatic	3.76	3.43	5.15	4.09	−2.70	0.007	5166	0.009
17. Social Problems	3.88	3.19	4.66	4.16	−1.53	0.127	5974	0.326
18. Thought Problems	4.62	4.19	6.32	4.89	−2.75	0.007	5103	0.006
19. Attention Problems	5.66	3.43	6.08	3.34	−0.94	0.351	5888	0.249
20. Rule Breaking	5.62	4.89	7.44	5.41	−2.60	0.010	5149	0.009
21. Aggressive	8.20	5.14	10.12	5.90	−2.55	0.011	5111	0.007

Legend: M = mean; SD = standard deviation; t = t statistic; U = U statistic; *p* = *p*-value.

**Table 4:** Pearson’s Correlations Between Risk-Taking and Self-Harm Behaviors and Other Psychological Variables.

Variable	1	2	3	4	5	6	7	8	9	10	11	12	13	14	15	16	17	18	19	20
1. Risk Taking	—																			
2. Self-Harm	0.33 ***	—																		
3. Risk Assessment	0.34 ***	0.20 **	—																	
4. Risk-Propensity	0.40 ***	0.15 *	0.35 ***	—																
5. Non-Acceptance	0.16 *	0.38 ***	0.16 *	0.03	—															
6. Goals	0.07	0.39 ***	0.15 *	0.05	0.54 ***	—														
7. Strategies	0.26 ***	0.58 ***	0.30 ***	0.14 *	0.53 ***	0.53 ***	—													
8. Impulse	0.32 ***	0.39 ***	0.32 ***	0.26 ***	0.33 ***	0.45 ***	0.53 ***	—												
9. Clarity	0.09	0.37 ***	0.24 ***	0.19 **	0.34 ***	0.36 ***	0.57 ***	0.30 ***	—											
10. Awareness	0.17 *	0.21 **	0.37 ***	0.12	0.17 **	0.10	0.35 ***	0.19 **	0.46 ***	—										
11. Security	−0.20 **	−0.39 ***	−0.24 ***	−0.13 *	−0.23 ***	−0.24 ***	−0.54 ***	−0.28 ***	−0.41 ***	−0.35 ***	—									
12. Anxiety	0.00	0.26 ***	0.02	0.06	0.35 ***	0.33 ***	0.40 ***	0.28 ***	0.27 ***	−0.05	−0.13	—								
13. Avoidance	0.15 *	0.38 ***	0.19 **	0.08	0.43 ***	0.37 ***	0.46 ***	0.29 ***	0.46 ***	0.32 ***	−0.54 ***	0.36 ***	—							
14. Anxious/Depressed	0.19 **	0.52 ***	0.15 *	−0.04	0.43 ***	0.40 ***	0.49 ***	0.32 ***	0.36 ***	0.19 **	−0.33 ***	0.44 ***	0.36 ***	—						
15. Withdrawn	0.16 *	0.52 ***	0.15 *	−0.04	0.40 ***	0.39 ***	0.51 ***	0.21 **	0.43 ***	0.23 ***	−0.42 ***	0.32 ***	0.46 ***	0.72 ***	—					
16. Somatic	0.28 ***	0.53 ***	0.17 **	0.00	0.34 ***	0.32 ***	0.44 ***	0.29 ***	0.35 ***	0.21 **	−0.32 ***	0.31 ***	0.34 ***	0.73 ***	0.60 ***	—				
17. Social Problems	0.26 ***	0.57 ***	0.26 ***	0.04	0.43 ***	0.34 ***	0.50 ***	0.34 ***	0.37 ***	0.31 ***	−0.48 ***	0.22 ***	0.43 ***	0.73 ***	0.72 ***	0.64 ***	—			
18. Thought Problems	0.47 ***	0.59 ***	0.25 ***	0.20 **	0.40 ***	0.28 ***	0.47 ***	0.36 ***	0.33 ***	0.21 **	−0.32 ***	0.26 ***	0.35 ***	0.67 ***	0.60 ***	0.62 ***	0.63 ***	—		
19. Attention Problems	0.35 ***	0.34 ***	0.38 ***	0.27 ***	0.18 **	0.30 ***	0.44 ***	0.36 ***	0.34 ***	0.28 ***	−0.40 ***	0.18 **	0.28 ***	0.55 ***	0.54 ***	0.49 ***	0.55 ***	0.54 ***	—	
20. Rule Breaking	0.69 ***	0.35 ***	0.38 ***	0.38 ***	0.14 *	0.12	0.29 ***	0.35 ***	0.13 *	0.21 **	−0.27 ***	−0.02	0.20 **	0.34 ***	0.35 ***	0.38 ***	0.41 ***	0.58 ***	0.59 ***	—
21. Aggressive	0.57 ***	0.37 ***	0.39 ***	0.31 ***	0.22 ***	0.19 **	0.31 ***	0.42 ***	0.18 **	0.23 ***	−0.24 ***	0.10	0.22 ***	0.48 ***	0.47 ***	0.46 ***	0.55 ***	0.62 ***	0.60 ***	0.74 ***

N = 234 (* *p* < 0.05, ** *p* < 0.01, *** *p* < 0.001).

**Table 5:** Centrality Indices and Spinglass Community Membership for Variables in the Risk-Taking and Self-Harm Network.

Measure	Strength	Betweenness	Closeness	Community
1. Risk Taking	0.77	0	2.88	1
2. Self-Harm	0.76	**33**	**4.11**	2
3. Risk Assessment	0.71	14	3.08	3
4. Risk-Propensity	0.64	5	2.99	3
5. Non-Acceptance	0.76	10	3.14	3
6. Goals	0.82	1	2.88	3
7. Strategies	**1.29**	**51**	**4.17**	3
8. Impulse	0.79	6	3.44	3
9. Clarity	0.85	17	3.65	2
10. Awareness	0.68	15	3.31	3
11. Security	0.79	15	3.59	3
12. Anxiety	0.65	2	3.53	2
13. Avoidance	0.83	5	3.32	2
14. Anxious/Depressed	**1.33**	24	3.78	2
15. Withdrawn	1.04	6	3.45	2
16. Somatic	0.8	0	3.25	2
17. Social Problems	1.04	3	3.49	1
18. Thought Problems	1.04	**35**	**4.14**	1
19. Attention Problems	0.96	8	3.21	1
20. Rule Breaking	**1.29**	21	3.24	1
21. Aggressive	0.99	23	3.47	1

Note. The three most central nodes according to each index are reported in bold. The closeness values are multiplied by 1000.

## Data Availability

All data generated or analyzed during this study are included in this published article as electronic Supplementary Materials.

## Supplementary Materials

The following supporting information can be downloaded at: https://data.mendeley.com/datasets/zdxh36vxh3/1 (accessed on 25 August 2023). File S1: Cerniglia, Luca (2023), “Data set”, Mendeley Data, V1.
